# Impact of Cooking on Starch Digestibility in Foxtail Millets

**DOI:** 10.3390/foods13243983

**Published:** 2024-12-10

**Authors:** Xiaojiao Cheng, Yujue Wang, Simeng Li, Shiqing Huang, Shujun Wang

**Affiliations:** 1 State Key Laboratory of Food Nutrition and Safety, Tianjin University of Science & Technology, Tianjin 300457, China; 13234160723@163.com (X.C.); yjwang0507@outlook.com (Y.W.); 15904227476@163.com (S.L.); sqhuang2019@163.com (S.H.); 2 School of Food Science and Engineering, Tianjin University of Science & Technology, Tianjin 300457, China

**Keywords:** foxtail millets, cooking, in vitro digestibility, starch–lipid complex, total dietary fiber

## Abstract

While the digestibility of millet starch has been studied considerably, the effects of cooking on starch digestibility in millet remain insufficiently understood. This study investigated the effects of cooking on in vitro enzymatic starch digestion in eight cooked millet flour cultivars by seeking its correlations with the changes in composition (moisture, total starch, protein, lipids, total dietary fiber, and phenolics), structure, and physicochemical properties. Compared to raw flours, cooked flours had a similar content of total starch and protein, a lower content of moisture, lipids, and total phenolic content, and a higher content of total dietary fiber. Cooking disrupted starch granules and crystalline structures in all eight millets, promoting the formation of starch–lipid complexes and reducing the paste viscosity (except for CS07). The in vitro starch digestion of eight millet flours were lower than that of rice flour. Correlation analysis results revealed that in vitro starch digestibility in cooked millet flours was related to the amounts of starch–lipid complexes and the total dietary fiber content. These findings demonstrated that interactions between starch and other components during cooking are the key determinants for the digestion of starch in cooked foods.

## 1. Introduction

Millet is the 6th most cultivated cereal globally [1], encompassing varieties such as pearl millet (*Pennisetum glaucum*), foxtail millet (*Setaria italica*), proso millet (*Panicum miliaceum*), finger millet (Eleusine coracana), and common millet (Panicum miliaceum) [2]. Among these, foxtail millet stands out as the dominant variety in China, with its domestication and use as a grain crop dating back approximately 8700 years in the Yellow River basin [3,4]. Foxtail millet, recognized for its exceptional drought resistance, is vital for maintaining food security in the arid and semi-arid areas of Asia and Africa [3]. Nutritionally, millets contain as much as 65~75% dietary carbohydrates, 7~12% protein, 2~5% fat, 15~20% dietary fiber, 2~4% minerals, and small amounts of phytochemicals, making it an attractive cereal for health-conscious consumers [5,6].

Millets and millet-based foods can more effectively control postprandial blood glucose levels compared with other cereals, thereby having greater potential in reducing the risk of type II diabetes [7,8,9,10]. Starch is the predominant component of millet, and its digestibility play an important role in regulating postprandial blood glucose and insulin responses [11,12,13]. Slowly digested and absorbed starch has been linked to reduced risk of type II diabetes [2,14]. Therefore, understanding the digestibility of millet starch is of considerable significance.

There are many factors influencing the in vitro starch digestibility of millet, including the structure of starch, the presence of protein, lipids, polyphenols, and fibers in the granules, starch–protein or starch–lipid interactions [5,15,16]. Studies have shown that the presence of pores or pinholes in the millet starch granules facilitated the entry of starch hydrolyzing enzymes into the starch granules [17]. Endogenous lipids in millet were proposed to effectively reduce the starch hydrolysis rate by interacting with starch to form starch–lipid complex [18,19,20]. Additionally, proteins can form a barrier around starch granules or interact with starches to inhibit their enzymatic hydrolysis [21,22]. Phenolic compounds from millet seed coats, such as protocatechuic acid, gentisic acid, caffeic acid, vanillic acid, and ferulic acid have been shown to inhibit the activity of α-glucosidase and/or pancreatic α-amylase, potentially reducing starch digestibility and postprandial hyperglycemia [23,24,25]. While some studies demonstrated that millet fibers reduce starch digestibility by increasing the viscosity of the digestion mixture [15], further research is needed to confirm this.

Since millet is commonly consumed after thermal processing, starch digestibility in cooked millet warrants greater attention. Heating treatment alters the structural and functional properties of millet starch and its main components, potentially affecting starch digestibility. While the factors that influence millet starch digestibility have been well reviewed in the previous study [5], there remains a knowledge gap regarding how cooking-induced changes in millet structure and composition affect its starch digestion. Specifically, the relationship between these changes and starch digestibility has not been comprehensively explored. Understanding this information could benefit the development of millet and millet-based foods with desired digestibility. Therefore, the objective of this study is to investigate how thermal processing affects the structural and functional properties of starch and other key components in millet and to determine their relationship with starch digestibility in cooked foxtail millet. In this study, cooking was chosen as the thermal processing method, and eight foxtail millet cultivars used in our previous study [26] were taken as the research objects.

## 2. Materials and Methods

### 2.1. Materials

Eight foxtail millet cultivars (QH02, JG40, JG22, JG16, JG14, CS07, CN44, and CN35) were used in this study according to our previous study [26]. Xiaozhan rice gain (RF, Ninghe District, Tianjin City), used for comparison, was purchased in supermarket. All the harvested grains were collected in 2017 and stored at 4 °C before analysis. Glucose oxidase/peroxidase (GOPOD) kit and amyloglucosidase were purchased from Megazyme International Ireland Ltd. (Bray Co., Wicklow, Ireland). Amylose (A0512) and amylopectin (A8515), α-amylase (EC 3.2.1.1, type VI-B from porcine pancreas, 13 units/mg), pepsin (P7012, ≥2500 units/mg) were purchased from Sigma Chemical Co. (St. Louis, MO, USA). Other chemical reagents were all of analytical grades.

### 2.2. Preparation of Raw and Cooked Flours

The millets were ground into powder by a high-speed grinder (DJ-350, Deqing Baijie Electric Appliance Co., Ltd., Huzhou, China), and passed through 100 mesh nylon meshes to obtain the raw flours. Cooked flours were prepared according to [27]. Briefly, millet and rice grains were weighed into an aluminum canister and distilled water was added to obtain a ratio of 1:1.5 (*W*/*V*). Subsequently, the canisters were covered with aluminum foil, placed in a steamer, and steamed for 25 min. The cooked grains were freeze-dried, freeze-ground, and passed through 100 mesh nylon meshes to obtain the cooked flours.

### 2.3. Composition Analysis of Millet Flours

Moisture, protein, and fat analyses were carried out according to AOAC (2005). Total dietary fiber (TDF) content was measured using enzymatic–gravimetric methods according to AOAC (1997). The extraction and determination of polyphenols were performed according to a previous method [28]. The amount of total phenolic content was calculated from the standard calibration curve of gallic acid and expressed as mg gallic acid equivalents/g dry weight basis of sample (mg GAE/g DWB).

### 2.4. Morphological Observation

Morphology of millet flour samples were examined under a scanning electron microscope (SEM) (JSM-IT300LV, JEOL, Tokyo, Japan) according to the method of Qi et al. (2020) [26].

### 2.5. X-Ray Diffraction

Relative crystallinity of millet flours was determined using an X-ray diffractometer (D8 Advance, Bruker, Germany) according to the method reported previously [26]. Millet flours were equilibrated over a saturated NaCl solution for one week before scanning (4°~40° (2θ), 2°/min, step size of 0.02°).

### 2.6. Differential Scanning Calorimetry (DSC)

Thermal properties of millet flours were measured using a 200 F3 Differential Scanning Calorimeter (Netzsch, Germany). 3 mg of the flours and 9 μL of distilled water (1:3, *w*/*w*) were placed in sealed aluminum pans. The pans were sealed, equilibrated overnight at room temperature and heated from 20 °C to 120 °C at a rate of 10 °C/min. An empty aluminum pan served as the reference. The thermodynamic parameters were obtained through data recording software.

### 2.7. Rapid Viscosity Analysis

Pasting properties of millet flours were evaluated using an RVA-4 Rapid Viscosity Analyzer (Perten Instruments Australia, Macquarie Park, NSW, Australia) according to previous study [26]. Briefly, millet flours (3.0 g, dry basis) were mixed with 25 mL of distilled water in a canister. The mixtures were stirred initially with the plastic paddle and then put into the instrument under standardized protocols.

### 2.8. In Vitro Starch Digestibility

In vitro digestion of starch in cooked millet flours was analyzed using a gastric–intestinal digestion protocol according to a previous procedure [27] with some modifications. Flours (containing 100 mg starch, dry weight basis) were dispersed in 5 mL of pepsin solution (1 mg/mL in 0.01 M HCl). The flour suspension was incubated at 37 °C with continuous agitation (260 rpm) for 30 min. The suspension was neutralized using 5 mL of 0.01 M NaOH to stop enzyme reactions and then mixed with 25 mL of sodium acetate buffer (pH 6, 0.2 M). Subsequently, 5 mL of freshly prepared enzyme solution containing porcine pancreatic α-amylase (1645 U) and amyloglucosidase (41 U) was added to the above solution and incubated at 37 °C with continuous stirring (260 rpm) for 120 min. Aliquots (0.05 mL) collected at specified intervals and mixed with 0.95 mL of 95% ethanol were to stop the enzyme reaction. After centrifugation (13,000× *g*, 3 min), the glucose content in the supernatant was measured by the GOPOD kit according to method reported previously [26]. Starch digestograms were fitted to the first order kinetic equation:

$$C_{t}=1-e^{-kt}$$

where *C_t_* is the amount of starch digested at time *t* (min) and *k* (min^−1^) is the first-order rate coefficient.

### 2.9. Statistical Analysis

All experiments excluding XRD measurements were duplicated. The data shown are the mean values and standard deviations. Pearson correlation analysis and one-way analysis of variance (ANOVA) by Duncan’s test (*p* < 0.05) were conducted using the SPSS 19.0 (Chicago, IL, USA).

## 3. Results

### 3.1. Chemical Composition of Millet Flours

The composition of raw and cooked flours is shown in Table 1. The moisture content of cooked flours was lower than the corresponding raw flours due to the greater loss of water by freeze-drying. There were small differences in starch and protein content between raw and cooked flours. However, the lipid content decreased greatly after cooking, presumably due to a proportion of free lipids interacting with leached amylose to form starch–lipid complexes in which the lipids were present in the bound state and could not be measured. This result was confirmed by the XRD and DSC analyses. The content of TDF of raw millet flours varied from 4.12% to 9.96%. After cooking, the content of TDF of millet flours increased (except for JG14, CS07, and CN35), which was attributed to the formation of starch–lipid complex that was considered dietary fiber. The content of the TDF of cooked flour (JG14, CS07, and CN35) was lower than that of the corresponding raw flour which may be due to the thermal degradation of the unstable dietary fiber. The total phenolic content of raw millet flours varied from 5.77 to 7.11 mg GAE/g, which was similar to the previous study [29]. Cooking leads to the decrease in the total phenolic content of millet flours, consistent with the results reported previously [30,31], which was due to the thermal degradation of phenolic compounds, or some phenolic compounds that formed complexes with proteins and other macromolecules, thus reducing the extractability of the phenolics [30].

### 3.2. Crystalline Structure

**Figure 1:**
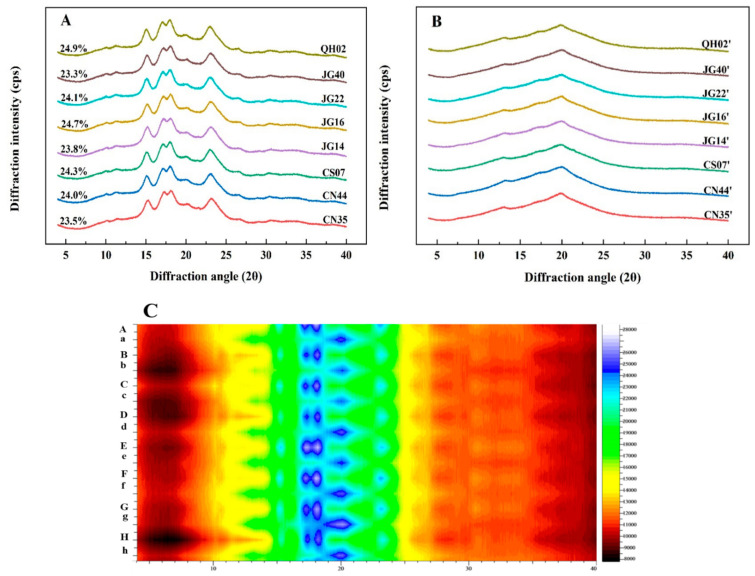
XRD patterns of raw and cooked flours: (**A**) raw flours; (**B**) cooked flours; (**C**) 2D views of XRD diffraction patterns of raw and cooked flours. A: QH02; B: JG40; C: JG22; D: JG16; E: JG14; F: CS07; G: CN44; H: CN35; a: QH02; b: JG40; c: JG22; d: JG16; e: JG14; f: CS07; g: CN44; h: CN35. Uppercase letters are raw flours, lowercase let-ters are cooked flours.

The XRD patterns of raw and cooked millet flours are shown in Figure 1A and Figure 1B, respectively. Raw flours showed typical diffraction peaks of A-type starch with two singlets at 15° and 23° (2θ) and an unresolved doublet at 17° and 18° (2θ). The relative crystallinities of eight raw millet flours were in the range of 23.3%~24.9%. After cooking, the typical diffraction patterns of A-type starch disappeared, indicative of starch being gelatinized. Two weak peaks at 13° and 20° (2θ) from V-type crystallinities were observed, indicating starch–lipid complexes were formed in cooked millet flours. Similar results were also observed with rice or wheat after cooking in previous studies [27,32].

To further illustrate the effect of cooking on the crystallinity of starch in millet, the two-dimensional views of X-ray diffraction pattern were generated (Figure 1C). The color scale on the right side of Figure 1C represented the peak intensity gradually increasing from black (bottom) to white (top). The blue color representing the A-type characteristic diffraction peaks (15°, 17°, 18°, 23°) of starch in raw millet flour was invisible after cooking. However, the yellow color at 13° and the green color at 20° (2θ) changed to a green color and a blue color, respectively, further demonstrating that starch–lipid complexes were formed in cooked millet flours. Similar results were also observed with wheat flours after cooking [32].

### 3.3. Thermal Properties

The thermal transition parameters of raw and cooked millet flours are displayed in Table 2 and Table 3, respectively. Eight raw millet flours exhibited a typical gelatinization endotherm in the range of 67.2~82.4 °C with the gelatinization enthalpy change (ΔH) ranging from 7.23 to 8.78 J/g. The weak endothermic transitions in the range of 84.9~102.3 °C were observed for all flours, indicating the presence of starch–lipid complexes in raw flours [32].

Cooked millet flours did not show a gelatinization endotherm of starch, indicating the complete disruption of starch crystallites during cooking. However, an endotherm with T_p_ of 94.7~97.1 °C and ΔH values of 1.3~2.5 J/g was observed for all cooked millet flours. The greater ΔH_c_ values of cooked flours relative to raw flours were indicative of the further formation of starch–lipid complexes during cooking, consistent with the XRD results. The different increases in ΔH values between raw and cooked flours suggested the complexing ability between starch and lipids differed greatly in different flours.

### 3.4. Pasting Properties

**Figure 2:**
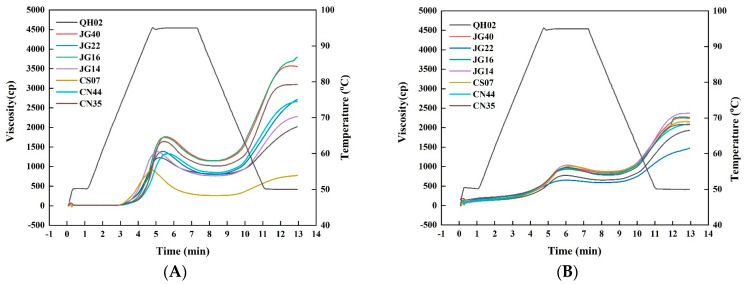
Pasting profiles of raw and cooked flours: (**A**) raw flours; (**B**) cooked flours.

The RVA pasting profiles of raw and cooked millet flours are shown in Figure 2A and Figure 2B, respectively, with the corresponding viscosity parameters listed in Table 4. The peak (PV), trough (TV), breakdown (BD), final (FV), and setback (SB) viscosities of raw millet flours ranged from 908 to 1761 cP, 262 to 1156 cP, 419 to 646 cP, 777 to 3791 cP, and 515 to 2635 cP, respectively. Pasting temperatures (PT) of the raw flours were in the range of 76.7 °C to 86.8 °C. There were significant differences in pasting parameters between raw millet flours, with CS07 presenting the much lower values of PV, TV, FV, and PT compared to other flours.

All cooked millet flours, except CS07, showed decreased paste viscosities and increased pasting temperature (Figure 2B and Table 4) compared to the corresponding raw flours, indicating the disruption of starch crystallites during heat-induced gelatinization, in agreement with previous reports [27,33]. Despite of the complete disruption of starch crystallites in cooked millet flours, these flours still showed characteristic RVA pasting profiles, which was attributed to the residual short-range molecular order in gelatinized starch still having the potential to swell to form pastes [34].

### 3.5. In Vitro Starch Digestibility of Cooked Millet Flours

**Figure 3:**
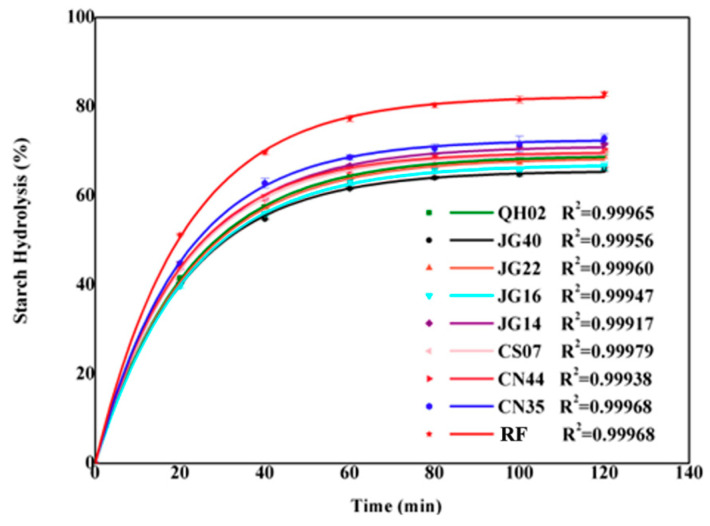
In vitro starch digestion curves of raw and cooked flours.

The in vitro enzymatic digestograms of starch in cooked millet flours are shown in Figure 3, with RDS, SDS, RS, and k calculated from digestograms listed in Table 5. Cooked rice flour prepared using the same method for cooked millet flour was used as the reference. As no glucose was detected during 30 min of simulated gastric digestion, the data were not displayed in the digestograms. Digestograms of cooked millet flours were characterized by a rapid digestion phase during the initial 40 min of incubation, followed by a slower hydrolysis stage before reaching the plateau at about 100 min (Figure 3). The RDS, SDS, and RS contents of eight cooked millet flours were in the range of 39.8~44.8%, 25.4~28.2% and 27.1~34.1%, respectively. In contrast, the RDS, SDS, and RS contents of cooked rice flours were 51.3%, 31.6%, and 17.1%, respectively. The k value of cooked millet flours ranged from 0.045 to 0.050 min^−1^, which was significantly lower than that of cooked rice flour (0.052 min^−1^). The above results show that starch in cooked millet was digested more slowly than that of starch in cooked rice.

## 4. Discussion

To identify the key factors determining the in vitro starch digestibility of eight cooked millet flours, Pearson correlation analyses between digestive properties and physicochemical properties of cooked flours were performed (Table 6). The enthalpy change in starch–lipid complex (ΔH_c_) was significantly positively correlated with RS content (r = 0.848, *p* < 0.01) and negatively correlated with RDS content (r = −0.806, *p* < 0.05) and k values (r = −0.752, *p* < 0.05). This result indicates that the formation of starch–lipid complexes during cooking, evidenced by the DSC and XRD results, significantly contribute to reduced starch susceptibility to enzymatic hydrolysis. Previous studies have explored the digestive properties of starch–lipid complexes and highlighted their potential benefits as slowly digestible and resistant starches [35,36,37]. Both the quantity of complexes and the quality of their stacking into V-type crystallites determined the digestion rate and extent of complexes [38,39]. Cooking increased the TDF content in most millet cultivars, likely due to the transformation of starch–lipid complexes into the dietary fiber fraction. Total dietary fiber content was significantly positively correlated with RS content (r = 0.919, *p* < 0.01) and negatively correlated with RDS (r = −0.793, *p* < 0.05). This result suggests that the increased dietary fiber induced by starch–lipid complex formation can inhibit enzymatic hydrolysis of starch. By integrating these correlation analyses, we proposed that the total dietary fiber and the amounts of starch–lipid complexes formed during cooking was the key factor in slowing down the digestion of starch in millet flours. Taken together, these findings illustrate how cooking affects the digestibility of starch in millets, which is of significance for optimizing the cooking parameters to modulate the digestibility of starch.

## 5. Conclusions

The present study demonstrated that cooking had little impact on the content of total starch and protein of millet flours but led to reductions in the content of lipid, total phenolics, and the paste viscosity (except for CS07). Conversely, cooking increased the total dietary fiber content. In addition, cooking resulted in the destruction of starch granule and crystalline structure of eight millets, promoting the formation of starch–lipid complexes. The in vitro starch digestibility of all millet flours was slower than that of the reference (rice flour). Correlation analysis indicated that the reduced in vitro starch digestibility in cooked millet flours was related to the formation of starch–lipid complexes and the increased total dietary fiber content. These findings provide valuable insights into the mechanisms that influence enzymatic starch digestion in cooked millet flours, which have significant implications in the field of millet-based food processing.

## Figures and Tables

**Table 1:** Moisture, total starch, protein and lipid content of raw and cooked flours.

Sample	Moisture (%)	Total Starch (%, Dry Basis)	Protein (%, Dry Basis)	Lipid (%, Dry Basis)	Total Dietary Fiber (%, Dry Basis)	Total Phenolic Content (mg GAE/g DWB)
Raw flours						
QH02	8.13 ± 0.06 a	71.94 ± 0.35 d	13.34 ± 0.02 e	3.26 ± 0.01 b	5.69 ± 1.09 bc	6.64 ± 0.17 c
JG40	9.77 ± 0.10 d	68.36 ± 0.39 b	12.68 ± 0.19 c	4.75 ± 0.47 d	4.12 ± 0.29 a	7.11 ± 0.08 e
JG22	9.71 ± 0.02 d	70.36 ± 0.53 c	10.94 ± 0.13 a	3.92 ± 0.05 c	5.19 ± 0.28 ab	5.77 ± 0.23 a
JG16	9.99 ± 0.04 e	69.03 ± 0.31 b	12.14 ± 0.05 b	4.76 ± 0.03 d	7.68 ± 0.66 d	6.16 ± 0.20 b
JG14	10.59 ± 0.13 f	70.52 ± 1.29 c	12.49 ± 0.02 c	2.50 ± 0.04 a	6.50 ± 0.90 bcd	5.98 ± 0.13 ab
CS07	8.52 ± 0.07 b	68.27 ± 0.48 b	12.25 ± 0.12 b	4.02 ± 0.04 c	9.96 ± 0.74 e	7.01 ± 0.29 de
CN44	8.83 ± 0.02 c	66.67 ± 0.56 a	13.24 ± 0.10 de	4.66 ± 0.14 d	6.92 ± 0.06 cd	6.76 ± 0.02 cd
CN35	11.08 ± 0.09 g	67.85 ± 0.25 b	13.05 ± 0.03 d	2.25 ± 0.02 a	7.40 ± 0.37 d	6.78 ± 0.12 cd
Cooked flours						
QH02	7.49 ± 0.03 e	67.98 ± 0.29 b	12.93 ± 0.21 ef	1.73 ± 0.02 b	7.79 ± 0.24 b	5.69 ± 0.05 de
JG40	6.56 ± 0.51 bcd	66.26 ± 0.18 a	12.48 ± 0.02 d	1.01 ± 0.33 a	10.58 ± 0.57 e	5.88 ± 0.27 e
JG22	5.74 ± 0.11 a	68.49 ± 0.20 b	10.45 ± 0.11 a	1.05 ± 0.21 a	9.55 ± 0.23 d	4.79 ± 0.12 a
JG16	6.72 ± 0.09 bcd	66.68 ± 0.27 a	12.08 ± 0.03 c	1.39 ± 0.18 ab	9.41 ± 0.08 d	5.42 ± 0.11 c
JG14	6.96 ± 0.02 d	69.61 ± 0.21 c	13.03 ± 0.04 f	1.16 ± 0.11 a	5.63 ± 0.03 a	4.83 ± 0.09 a
CS07	6.36 ± 0.12 b	66.78 ± 0.68 a	11.65 ± 0.02 b	1.40 ± 0.16 ab	8.40 ± 0.04 c	5.74 ± 0.06 e
CN44	6.37 ± 0.08 bc	68.23 ± 0.42 b	13.17 ± 0.16 f	1.09 ± 0.08 a	7.96 ± 0.09 bc	5.48 ± 0.10 cd
CN35	6.87 ± 0.26 cd	69.51 ± 0.70 c	12.69 ± 0.02 de	1.22 ± 0.05 a	6.09 ± 0.25 a	5.17 ± 0.07 b

Values are means ± SD. Statistical analysis was conducted separately for raw flours and cooked flours within each column. Different letters indicate the significant differences among cultivars with the same processing condition (raw or cooked) (*p* < 0.05).

**Table 2:** Thermal transition parameters of raw millet flours.

Sample	T_o1_ (°C)	T_p1_ (°C)	T_c1_ (°C)	ΔH_1_ (J/g)	T_o2_ (°C)	T_p2_ (°C)	T_c2_ (°C)	ΔH_c_ (J/g)
QH02	68.8 ± 0.1 c	74.2 ± 0.1 e	79.1 ± 0.00 cd	8.8 ± 0.2 e	85.4 ± 0.8 a	93.8 ± 0.1 a	99.7 ± 0.6 ab	1.1 ± 0.1 e
JG40	68.9 ± 0.1 c	73.7 ± 0.00 d	78.6 ± 0.3 bc	7.2 ± 0.2 a	90.2 ± 0.9 d	96.9 ± 0.5 de	100.4 ± 0.6 abc	0.4 ± 0.0 b
JG22	67.2 ± 0.3 b	72.6 ± 0.1 b	79.0 ± 0.1 cd	8.0 ± 0.1 c	88.7 ± 0.1 bc	94.8 ± 0.1 b	100.6 ± 0.0 abc	0.9 ± 0.1 d
JG16	70.3 ± 0.4 d	74.9 ± 0.1 f	82.4 ± 0.2 g	8.5 ± 0.1 de	89.6 ± 0.0 cd	96.5 ± 0.5 cde	102.3 ± 0.6 c	0.1 ± 0.0 a
JG14	69.4 ± 0.1 c	75.1 ± 0.1 f	80.1 ± 0.1 e	8.4 ± 0.1 d	92.0 ± 0.1 e	95.6 ± 0.1 bc	99.2 ± 0.1 a	0.4 ± 0.0 b
CS07	69.1 ± 0.1 c	73.4 ± 0.1 c	78.1 ± 0.1 b	7.6 ± 0.1 b	93.4 ± 0.1 f	97.3 ± 0.1 e	102.3 ± 0.5 c	1.1 ± 0.0 e
CN44	69.1 ± 0.1 c	76.4 ± 0.1 g	80.7 ± 0.1 f	7.4 ± 0.1 ab	88.2 ± 0.2 b	95.6 ± 0.1 bc	99.3 ± 0.1 ab	0.8 ± 0.0 c
CN35	68.9 ± 0.5 c	73.8 ± 0.2 d	79.4 ± 0.4 d	8.0 ± 0.2 c	84.9 ± 0.4 a	93.6 ± 0.6 a	100.6 ± 0.1 abc	0.8 ± 0.0 c

Values are means ± SD. Values with the different letters in a column are significantly different (*p* < 0.05). The transition temperature of starch, T_o1_: onset temperatures; T_p1_: peak temperatures; T_c1_: conclusion temperatures; ΔH_1_: enthalpy change in gelatinization; The transition temperature of starch–lipid complex, T_o2_, T_p2_, T_c2_; ΔH_c_: enthalpy change in starch–lipid complex.

**Table 3:** Thermal transition parameters of starch–lipid complexes in cooked millet flours.

Sample	T_o_ (°C)	T_p_ (°C)	T_c_ (°C)	ΔH_c_ (J/g)
QH02	86.9 ± 1.5 bc	96.0 ± 0.3 ab	100.8 ± 0.1 ab	2.2 ± 0.1 cd
JG40	85.0 ± 0.2 a	94.7 ± 0.6 a	100.6 ± 0.1 a	2.4 ± 0.1 ef
JG22	87.5 ± 0.6 c	96.1 ± 0.7 ab	101.1 ± 0.5 ab	2.5 ± 0.1 f
JG16	87.9 ± 0.6 c	96.2 ± 0.5 ab	101.0 ± 0.1 ab	2.3 ± 0.1 de
JG14	87.9 ± 0.9 c	96.2 ± 0.0 ab	100.5 ± 0.3 a	1.4 ± 0.1 a
CS07	86.7 ± 0.2 abc	97.1 ± 1.0 b	100.6 ± 0.3 a	2.1 ± 0.1 bc
CN44	86.7 ± 0.3 abc	97.0 ± 0.1 b	101.5 ± 0.1 b	2.0 ± 0.0 b
CN35	85.3 ± 0.9 ab	95.9 ± 2.0 ab	101.15 ± 0.6 ab	1.3 ± 0.1 a

Values are means ± SD. Values with the different letters in a column are significantly different (*p* < 0.05). T_o_: onset temperatures; T_p_: peak temperatures; T_c_: conclusion temperatures; ΔH_c_: enthalpy change in starch–lipid complex.

**Table 4:** Pasting parameters of raw and cooked flours.

Sample	PV (cP)	TV (cP)	BD (cP)	FV (cP)	SB (cP)	PT (°C)
Raw flours						
QH02	1222 ± 8.5 b	804 ± 4.9 bc	419 ± 3.5 a	2007 ± 16.3 b	1203 ± 11.3 b	79.5 ± 0.5 b
JG40	1740 ± 25.5 f	1129 ± 16.4 e	610 ± 11.5 cd	3512 ± 83.1 f	2382 ± 80.4 g	85.1 ± 0.4 d
JG22	1389 ± 0.7 d	798 ± 7.8 bc	591 ± 7.1 c	2701 ± 15.6 d	1904 ± 7.8 e	78.7 ± 0.5 b
JG16	1761 ± 17.7 f	1156 ± 8.2 e	605 ± 17.2 cd	3791 ± 23.5 g	2635 ± 27.4 h	82.0 ± 0.4 c
JG14	1397 ± 9.9 d	769 ± 4.2 b	628 ± 5.7 de	2289 ± 14.8 c	1520 ± 10.6 c	79.1 ± 0.0 b
CS07	908 ± 0.7 a	262 ± 2.1 a	646 ± 1.4 e	777 ± 2.1 a	515 ± 0.0 a	76.7 ± 0.0 a
CN44	1314 ± 19.1 c	823 ± 34.6 c	491 ± 15.6 b	2615 ± 74.2 d	1792 ± 39.6 d	86.0 ± 0.6 de
CN35	1640 ± 2.1 e	1038 ± 29.7 d	602 ± 31.8 cd	3088 ± 7.1 e	2050 ± 36.8 f	86.8 ± 0.6 e
Cooked flours						
QH02	767 ± 12.0 b	651 ± 9.2 b	116 ± 2.8 b	1922 ± 13.4 b	1271 ± 4.2 b	94.0 ± 0.6 e
JG40	987 ± 21.2 de	868 ± 21.9 e	120 ± 0.7 b	2259 ± 23.3 ef	1391 ± 1.4 cd	88.0 ± 0.1 a
JG22	640 ± 16.3 a	580 ± 12.0 a	60 ± 4.2 a	1451 ± 34.6 a	871 ± 22.6 a	91.2 ± 0.2 c
JG16	927 ± 10.6 c	813 ± 8.5 d	114 ± 2.1 b	2253 ± 17.7 ef	1440 ± 9.2 de	89.3 ± 0.6 b
JG14	1030 ± 12.0 f	859 ± 9.2 e	171 ± 2.8 d	2337 ± 48.1 f	1479 ± 38.9 e	90.5 ± 0.1 c
CS07	1014 ± 3.5 ef	874 ± 2.1 e	140 ± 5.7 c	2187 ± 51.6 de	1313 ± 53.7 bc	92.5 ± 0.6 d
CN44	967 ± 3.5 d	818 ± 7.8 d	149 ± 4.2 c	2143 ± 70.0 cd	1325 ± 62.2 bc	90.9 ± 0.6 c
CN35	985 ± 9.9 de	787 ± 3.5 c	199 ± 6.4 e	2092 ± 16.3 c	1305 ± 12.7 b	92.4 ± 0.6 d

Values are means ± SD. Values with the different letters in a column are significantly different (*p* < 0.05). PV, TV, BD, FV, SB, PT correspond to the peak, trough, breakdown, final, setback viscosity and pasting temperature, respectively.

**Table 5:** Starch fraction of cooked millet flours.

Samples	RDS (%)	SDS (%)	RS (%)	*k* (min^−1^)
QH02	41.6 ± 0.37 c	27.9 ± 0.30 cd	30.6 ± 0.27 de	0.046 ± 0.001 ab
JG40	40.5 ± 0.13 b	25.4 ± 0.68 a	34.1 ± 0.55 f	0.046 ± 0.001 bc
JG22	40.7 ± 0.30 b	28.2 ± 0.89 d	31.1 ± 0.71 e	0.045 ± 0.001 a
JG16	39.8 ± 0.46 a	26.9 ± 0.73 bc	33.3 ± 0.89 f	0.045 ± 0.001 ab
JG14	43.9 ± 0.46 de	27.6 ± 0.52 cd	28.4 ± 0.20 c	0.048 ± 0.001 bc
CS07	43.6 ± 0.19 d	26.2 ± 0.21 ab	30.3 ± 0.22 de	0.049 ± 0.000 d
CN44	44.5 ± 0.48 ef	25.9 ± 0.36 ab	29.6 ± 0.31 d	0.050 ± 0.001 d
CN35	44.8 ± 0.36 f	28.1 ± 0.62 d	27.1 ± 0.91 b	0.050 ± 0.001 d
RF	51.3 ± 0.23 g	31.6 ± 0.60 e	17.1 ± 0.45 a	0.052 ± 0.000 e

Values are means ± SD. Values with the different letters in a column are significantly different (*p* < 0.05). RDS, rapidly digested starch content; SDS, slowly digested starch content; RS, resistant starch content.

**Table 6:** Pearson correlation coefficients between digestive properties and other properties of eight cooked millet flours.

Sample	RDS	SDS	RS	*k*
ΔH_c_	−0.806 *	−0.325	0.848 **	−0.752 *
Protein	0.470	−0.218	−0.302	0.491
Lipid	−0.094	0.284	−0.039	−0.144
Total dietary fiber	−0.793 *	−0.498	0.919 **	−0.639
Total phenol	−0.172	−0.702	0.489	−0.018

Significant correlations are indicated in bold, with * *p* < 0.05 and ** *p* < 0.01.

## Data Availability

The original contributions presented in the study are included in the article, further inquiries can be directed to the corresponding author.
